# Vorinostat in the acute neuroinflammatory form of X‐linked adrenoleukodystrophy

**DOI:** 10.1002/acn3.51015

**Published:** 2020-05-02

**Authors:** Bettina Zierfuss, Isabelle Weinhofer, Jörn‐Sven Kühl, Wolfgang Köhler, Annette Bley, Katharina Zauner, Johannes Binder, Ksenija Martinović, Christian Seiser, Christoph Hertzberg, Stephan Kemp, Gerda Egger, Gerda Leitner, Jan Bauer, Christoph Wiesinger, Markus Kunze, Sonja Forss‐Petter, Johannes Berger

**Affiliations:** ^1^ Department of Pathobiology of the Nervous System Center for Brain Research Medical University of Vienna Vienna Austria; ^2^ Department of Pediatric Oncology, Hematology, and Hemostaseology University Hospital Leipzig Leipzig Germany; ^3^ Department of Neurology University of Leipzig Medical Center Leukodystrophy Clinic Leipzig Germany; ^4^ Department of Pediatrics University Medical Center Hamburg Eppendorf Hamburg Germany; ^5^ Division of Cell and Developmental Biology Center for Anatomy and Cell Biology Medical University of Vienna Vienna Austria; ^6^ Vivantes Klinikum Neukölln Berlin Germany; ^7^ Department of Genetic Metabolic Diseases Amsterdam UMC University of Amsterdam Amsterdam The Netherlands; ^8^ Department of Pathology Medical University of Vienna Vienna Austria; ^9^ Ludwig Boltzmann Institute Applied Diagnostics Vienna Austria; ^10^ Department of Blood Group Serology and Transfusion Medicine Medical University of Vienna Vienna Austria; ^11^ Department of Neuroimmunology Center for Brain Research Medical University of Vienna Vienna Austria

## Abstract

**Objective:**

To identify a pharmacological compound targeting macrophages, the most affected immune cells in inflammatory X‐linked adrenoleukodystrophy (cerebral X‐ALD) caused by *ABCD1* mutations and involved in the success of hematopoietic stem cell transplantation and gene therapy.

**Methods:**

A comparative database analysis elucidated the epigenetic repressing mechanism of the related *ABCD2* gene in macrophages and identified the histone deacetylase (HDAC) inhibitor Vorinostat as a compound to induce ABCD2 in these cells to compensate for ABCD1 deficiency. In these cells, we investigated *ABCD2* and pro‐inflammatory gene expression, restoration of defective peroxisomal β‐oxidation activity, accumulation of very long‐chain fatty acids (VLCFAs) and their differentiation status. We investigated *ABCD2* and pro‐inflammatory gene expression, restoration of defective peroxisomal ß‐oxidation activity, accumulation of very long‐chain fatty acids (VLCFA) and differentiation status. Three advanced cerebral X‐ALD patients received Vorinostat and CSF and MRI diagnostics was carried out in one patient after 80 days of treatment.

**Results:**

Vorinostat improved the metabolic defects in X‐ALD macrophages by stimulating *ABCD2* expression, peroxisomal ß‐oxidation, and ameliorating VLCFA accumulation. Vorinostat interfered with pro‐inflammatory skewing of X‐ALD macrophages by correcting *IL12B* expression and further reducing monocyte differentiation. Vorinostat normalized the albumin and immunoglobulin CSF‐serum ratios, but not gadolinium enhancement upon 80 days of treatment.

**Interpretation:**

The beneficial effects of HDAC inhibitors on macrophages in X‐ALD and the improvement of the blood‐CSF/blood‐brain barrier are encouraging for future investigations. In contrast with Vorinostat, less toxic macrophage‐specific HDAC inhibitors might improve also the clinical state of X‐ALD patients with advanced inflammatory demyelination.

## Introduction

X‐linked adrenoleukodystrophy (X‐ALD) is a neurodegenerative disease (OMIM #300100) caused by mutations in the *ATP‐binding cassette subfamily D member 1 (ABCD1)* gene, which encodes a peroxisomal transporter crucial for the import of coenzyme A‐activated very long‐chain fatty acids (VLCFAs) into peroxisomes for degradation.^1^, ^2^, ^3^ Accordingly, ABCD1 deficiency results in accumulation of VLCFAs in tissues and body fluids of patients.^4^ Cerebral ALD (CALD), the most severe form, affects ~60% of male X‐ALD patients and is characterized by a rapidly progressive inflammatory destruction of brain white matter.^5^, ^6^, ^7^ If untreated, CALD results in vegetative state or death within a few years after disease onset.^5^, ^6^, ^8^, ^9^ The inflammatory brain lesions are characterized by impaired integrity of the blood‐cerebrospinal fluid/blood‐brain barrier (BCSFB/BBB) and recruitment of immune cells from the periphery.^10^ If the onset is detected early, the inflammatory demyelination can be stopped by hematopoietic stem cell transplantation (HSCT) or gene therapy (HSCGT) without major disabilities.^11^, ^12^ However, HSCT/HSCGT have limited effect in more advanced patients. Both procedures may require up to 16 months to halt cerebral demyelination, and the necessary neurotoxic myeloablative chemo‐conditioning will further contribute to disease progression.^13^ Thus, for patients with advanced cerebral involvement (Loes score > 9) no effective treatment options are available.^14^ Pharmacological treatment of CALD may offer advantages in comparison to HSCT/HSCGT by having a lower mortality risk and immediate applicability of therapeutic effects.

Among different HSC‐derived immune cells, ABCD1 deficiency most severely affects monocytes/macrophages in terms of impaired VLCFA metabolism.^15^ Moreover, pro‐inflammatory skewed X‐ALD macrophages are less able to adopt an anti‐inflammatory state as shown in vitro and *postmortem*.^15^, ^16^ Thus, the presence of metabolically intact macrophages derived by HSCT/HSCGT appears to be key for the success in halting the inflammatory demyelination. One possibility to revert the metabolic defect in X‐ALD macrophages could be by pharmacological upregulation of the *ABCD2* gene.^17^ Upon overexpression, ABCD2 can compensate for ABCD1 deficiency in cultured cells and in *Abcd1*‐deficient mice.^18^, ^19^, ^20^, ^21^ However, in human monocytes/macrophages, *ABCD2* is barely expressed.^15^

Here, we compared the epigenetic marks at the human *ABCD2* locus of monocytes/macrophages and T cells (high *ABCD2* expression). Based on these results, we evaluated the therapeutic potential of the histone deacetylase (HDAC) inhibitor Vorinostat (Zolinza^®^, suberoylanilide hydroxamic acid, SAHA) for the neuroinflammation in CALD. Vorinostat, an anti‐cancer agent,^22^, ^23^ had positive effects on neuroinflammation in an animal model of inflammatory demyelination^24^ and significantly reduced the incidence of graft‐versus‐host disease after HSCT.^25^, ^26^ Vorinostat and other pan‐HDAC inhibitors like phenylbutyrate and valproic acid were previously suggested as treatment options in X‐ALD, because of improving X‐ALD related features in other ABCD1‐deficient cell types.^17^, ^21^, ^27^, ^28^, ^29^

Here, we thoroughly evaluated the properties of Vorinostat in vitro in macrophages derived from seven X‐ALD patients. Based on these positive observations, three boys with advanced CALD, who had been diagnosed too late for HSCT/HSCGT and were left without alternative therapeutic options received Vorinostat on compassionate use.

## Materials and Methods

### Patients and healthy volunteers

Upon obtained informed consent and approval by the Ethical Committee of the Medical University of Vienna (EK1462/2014), peripheral blood samples were drawn from 18 healthy volunteers and from seven X‐ALD patients with AMN. Patients’ details are described in Table S1. The accumulation of VLCFAs in plasma and leukocytes of AMN patients was confirmed by measuring the total amount of the fatty acids C26:0, C24:0, and C22:0 by GC–MS as described previously.^15^ Three childhood CALD patients with advanced disease progression received Vorinostat orally under a compassionate‐use label after written informed consent from the patients’ parents. Written informed consent to publish the medical data and MRI images of the three Vorinostat‐treated CALD patients was obtained from the patients’ parents.

### In vitro differentiation of human monocytes to macrophages and activation with LPS

For macrophage differentiation and polarization, CD14^+^ monocytes (1 × 10^6^ cells/well) were isolated and characterized by flow cytometry as described previously^15^, ^16^ and seeded in RPMI medium (Sigma‐Aldrich) containing 1% Penicillin/Streptomycin, 1% glutamine, 1% Fungizone, and 10% fetal calf serum, supplemented with either 50 ng/mL human recombinant GM‐CSF or M‐CSF (PeproTech) for 7 days. For *β*‐oxidation assays, M‐CSF‐differentiated macrophages were polarized into an anti‐inflammatory phenotype with 100 ng/ml IL‐4 (Novartis) and 10 ng/ml M‐CSF for 2 days. To analyse pro‐inflammatory cytokine gene expression, M‐CSF‐differentiated macrophages were stimulated with 100 ng/ml LPS (*E*.* coli* 055:B5, #L4005, Sigma) and treated with vehicle control or Vorinostat (#10009929, Cayman Chemicals) for 24 h. ELISA to measure secreted IL12p40 was described previously.^16^ To determine mean cell size and number of adherent or nonadherent cells, a CASY automated cell counter (Omni Life Sciences) was used. For detachment, adherent macrophages were incubated with 300 *µ*L Gibco™ TrypLE™ Select (10×) (Gibco, Life Technologies) for 15 min at 37°C shaking every few minutes and was gently removed with a cell scraper.

### RNA isolation and reverse transcription coupled‐quantitative PCR (RT‐qPCR)

RNA isolation and RT‐qPCR analysis were carried out as previously described.^15^, ^16^ In addition, the reference gene *RACK1* (NM_006098) was analysed for normalization purposes as indicated by using the gene‐specific primers and probe: forward 5′gccataccaaggatgtgctg‐3′, reverse 5′‐tggttggtcttcagcttgca‐3′, TexasRed‐cgcccaacagcagcaaccct‐BHQ2.

### *β*‐Oxidation of 1‐^14^C‐labeled fatty acids

Detached macrophages (1–2 × 10^6^ per condition) were washed and resuspended in sucrose buffer (250 mmol/L sucrose, 3 mmol/L imidazole, 1 mmol/L EDTA, pH 7.4 at 4°C). Protein levels were determined and used for normalization. *β*‐Oxidation of radio‐labeled fatty acids to acetate was carried out as described previously.^3^, ^15^

### Lipid analysis

C26:0‐lysophophatidylcholine was analyzed in differentiated macrophages following the protocol for dried blood spots as described previously.^30^

### Analysis of Chromatin Immunoprecipitation‐DNA Sequencing (ChIP‐Seq) data for different histone modifications

ChIP‐Seq datasets of monocytes/macrophages and T cells were retrieved from the International Human Epigenome Consortium (IHEC) data portal.^31^ The datasets were preselected by assay category “histone” and tissue “blood.” The consortia BLUEPRINT, CEEHRC (Canadian Epigenetics, Environment and Health Research Consortium), DEEP (Deutsches Epigenom Programm) and Roadmap provided data on the human *ABCD*2 locus in both T‐cells and monocytes/macrophages and, thus, were included in our comparative study. For further data analysis and visualization, the software Integrative Genomics Viewer was used.^32^ As reference genome, hg19 was selected for CEEHRC, DEEP, and Roadmap, and hg38 for BLUEPRINT.

### Immunohistochemical analysis of B cells in human *postmortem* brain tissue

CD20 and IgG staining was performed as described previously.^33^ The *postmortem* tissue of four CALD patients and six control cases were described in detail previously.^16^

### Statistical analysis

For normally distributed data, we performed one‐way ANOVA and Sidak’s or Dunnett’s multiple comparison test as indicated. For not normally distributed data, we used the nonparametric Mann–Whitney or Friedman tests and post hoc Dunn’s multiple comparison test. The *P*‐values were calculated and the null hypothesis was denied for *P*‐values <0.05. GraphPad Prism 7 (GraphPad Software) was used for graphical display and statistical analysis.

## Results

### The *ABCD2* gene is epigenetically repressed in monocytes and macrophages

**Figure 1:**
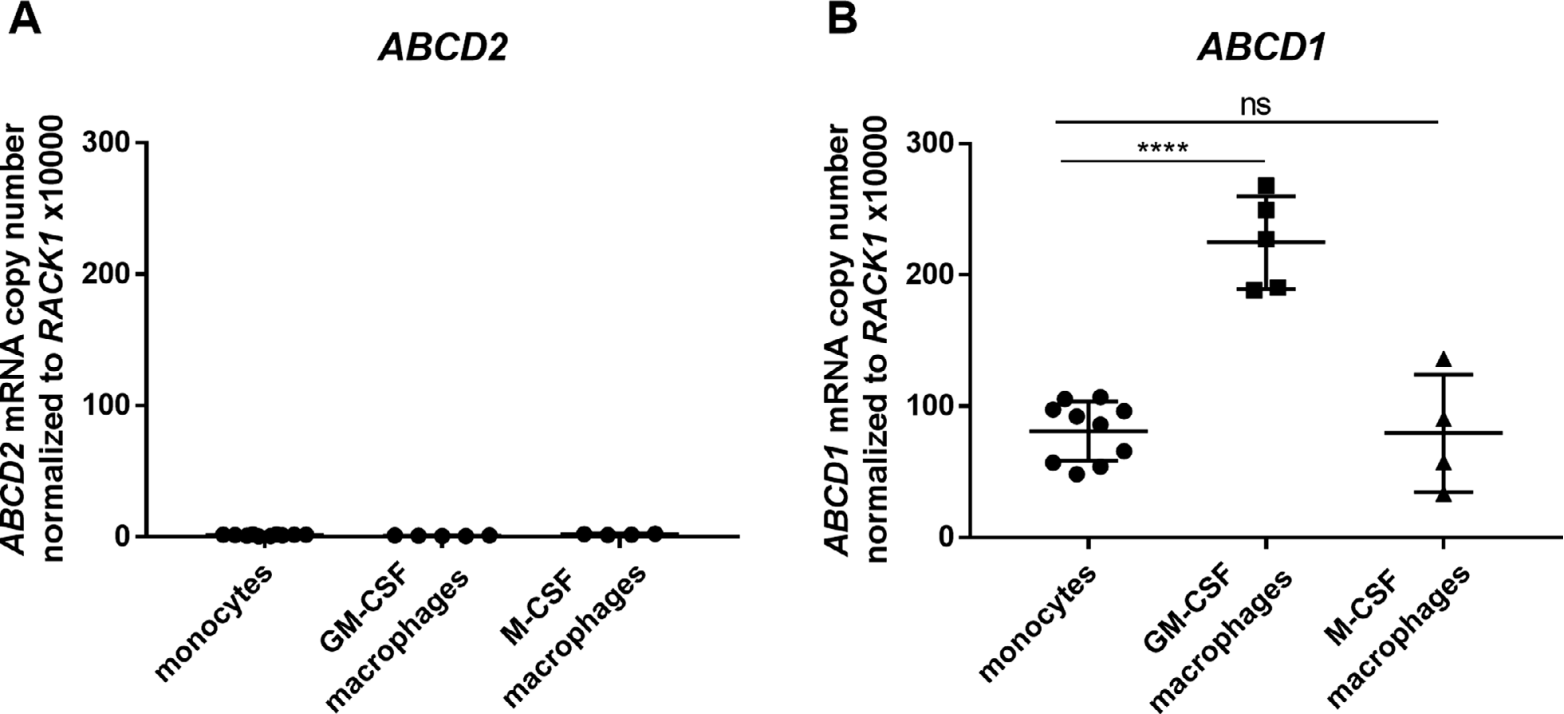
*ABCD2* gene expression remains at low levels compared to *ABCD1* after differentiation using GM‐CSF or M‐CSF in human monocyte‐derived macrophages. Monocytes were isolated from leukocyte concentrates derived from healthy donors by plasmapheresis and were differentiated toward macrophages with 50 ng/mL GM‐CSF or M‐CSF for 7 days. Absolute mRNA levels were determined by RT‐qPCR using standard concentration curves of plasmids containing (A) *ABCD2* and (B) *ABCD1* and for normalization *RACK1* cDNA. Each data point represents a replicate (well) from three healthy donors (controls 1–3). For statistical analysis one‐way ANOVA and Sidak’s multiple comparison test were used. ****P < 0.0001, *ns* = not significant.

Upon infiltrating the brain parenchyma, monocytes, which normally express *ABCD1* but barely *ABCD2*,^15^ differentiate into mature macrophages. Thus, we first investigated how differentiation of monocytes to macrophages affects *ABCD1* and *ABCD2* expression and found that also macrophages hardly express the *ABCD2* gene (Fig. 1A). In contrast, *ABCD1* was already highly expressed in monocytes and even further increased after differentiation with GM‐CSF, which is assumed to have pro‐inflammatory properties.^34^ Interestingly, upon differentiation with M‐CSF, a cytokine required for the maintenance of most macrophage populations at steady state,^34^ *ABCD1* expression did not differ from that of monocytes (Fig. 1B).

Unlike monocytes/macrophages, T cells highly express the *ABCD2* gene.^15^ In order to elucidate the underlying cause for the differential expression in these cell types, we next sought to identify potential epigenetic mechanisms that repress *ABCD2* in the macrophage lineage. Data retrieved from a recent study of the differential DNA methylation patterns in human purified blood cells^35^ indicated no major differences in DNA methylation within the *ABCD2* promoter region that would explain the large differential expression between T cells and monocytes. Based on the low density of CpG sites in the promoter and small differences in methylation levels, we assume that DNA methylation is not causally involved in the silencing of the *ABCD2* gene.

**Figure 2:**
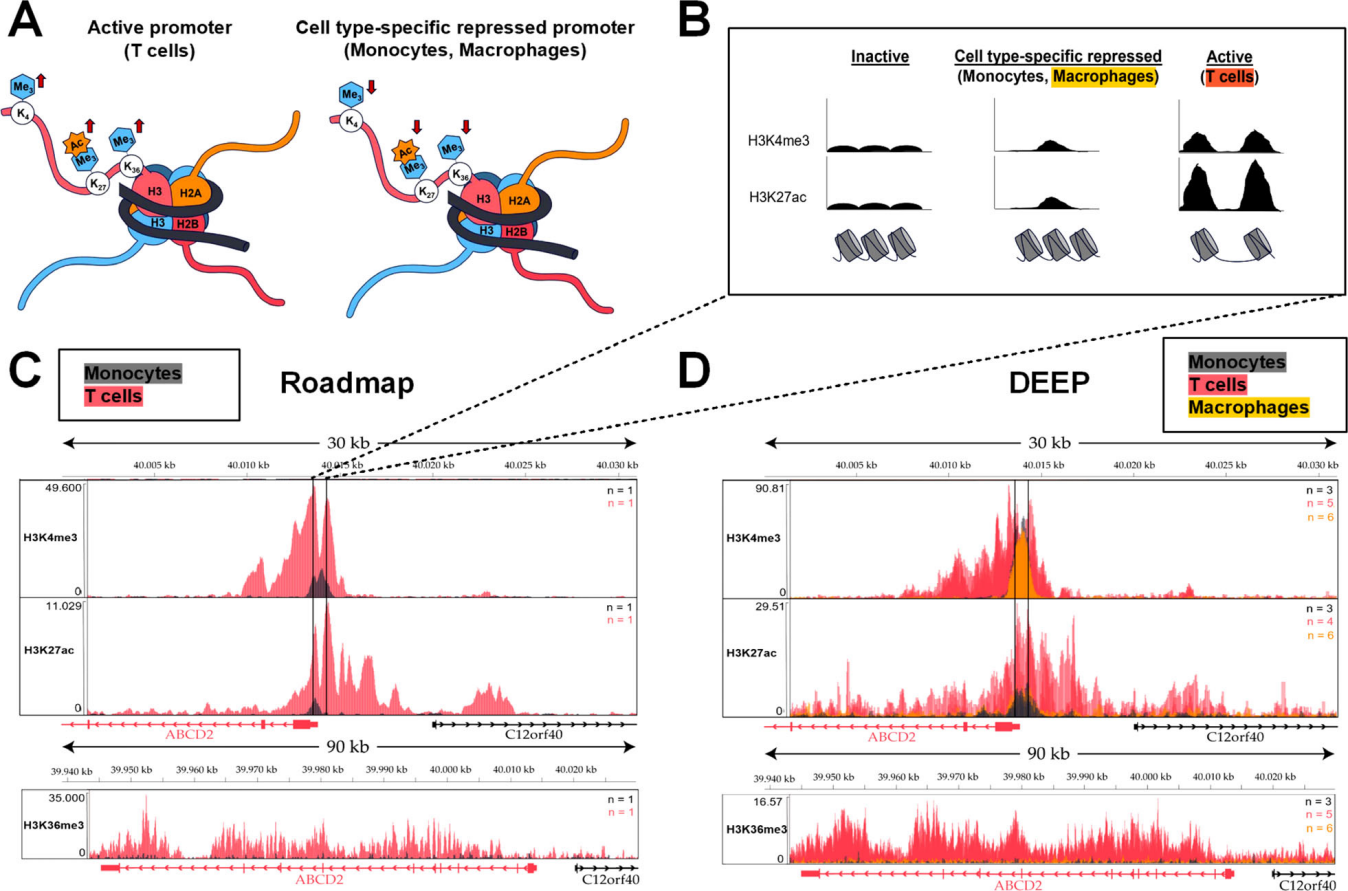
Comparison of activating histone modifications indicates a cell type‐specific repressed state of the *ABCD2* promoter in monocytes and macrophages. ChIP‐Seq data of activating histone modifications retrieved from the International Human Epigenome Consortium (IHEC) were compared in human monocytes (dark grey), macrophages (orange) and T cells (red) to analyse the mechanism of differential *ABCD2* expression, which is high in T cells but barely detectable in monocytes/macrophages. (A) An active promoter is characterized by high trimethylation of histone 3 lysine 4 (H3K4me3) and acetylation (ac) of H3K27. In addition, active expression of a gene is associated with high H3K36me3 marks at intragenic regions. The *vice versa* pattern could indicate a cell type‐specific, repressed state as indicated in the scheme. (B) Nucleosome‐free regions of DNA are easily accessible for transcription factors and the RNA‐polymerase II and, thus, depending on flanking histone modifications can be linked with an active or poised promoter. Accordingly, the analysis of ChIP‐Seq data reveals the associated characteristic peak‐valley‐peak pattern of H3K4me3 and H3K27ac at a promoter linked to active gene expression. However, a peak spanning the promoter region is associated with a repressed state, while low levels of these modifications typically indicate an inactivated state. (C, D) In the upper panel, a 30‐kb region (GRCh37; Chr 12: 40,000,000–40,031,000) surrounding the *ABCD2* promoter is depicted. Within the promoter region (marked with vertical lines) data from the consortia Roadmap (C) and DEEP (D) show a typical peak‐valley‐peak pattern for H3K4me3 and H3K27ac in T cells (red) indicating an active promoter. In monocytes (dark grey) and macrophages (orange), these modifications span a broader region around the promoter. The bottom panels show elevated H3K36me3 marks at intragenic regions of *ABCD2* in T cells, indicating active expression, but barely detectable signals in monocytes/macrophages in datasets from both consortia (C, D). *n* = number of tracks per cell type.

Besides DNA methylation, also histone modifications regulate gene expression. While promoter regions of actively expressed genes are characterized by high histone 3 lysine 4 tri‐methylation (H3K4me3), low histone 3 lysine 4 mono‐methylation (H3K4me1), and high histone 3 lysine 27 acetylation (H3K27ac), active enhancer regions show high H3K4me1 and H3K27ac modifications.^36^ In addition, expressed genes are marked by high levels of histone 3 lysine 36 tri‐methylation (H3K36me3), which are associated with active transcription by RNA‐polymerase II within gene body regions (Fig. 2A). Importantly, H3K4me3 and H3K27ac can occur in a peak‐valley‐peak (PVP) pattern at the promoter region due to a nucleosome‐depleted region allowing promoter accessibility for transcription factors and RNA‐polymerase II. In contrast, repressed but not inactivated promoters are often marked by low H3K4me3 and H3K27ac spanning the whole promoter region (Fig. 2B).^37^ Here, we compared ChIP‐Seq data sets from four different consortia that had been deposited at the IHEC^31^ and found similar epigenetic patterns of the *ABCD2* locus (Fig. 2C and D show exemplary data). As expected, H3K36me3, indicative of active expression, was high at intragenic regions of *ABCD2* in T cells but barely detectable in monocytes/macrophages (Fig. 2C and D). At the *ABCD2* promoter, T cells show a PVP of H3K4me3 and H3K27ac characteristic for active promoters. Monocytes/macrophages, however, harbor a broader single peak for H3K4me3 and H3K27ac modifications, centered at the position of the valley in the PVP of T cells, possibly indicating a cell type‐specific nucleosome positioning and repressed state of *ABCD2* (Fig. 2C and D).

### Vorinostat efficiently induces *ABCD2* expression and improves VLCFA metabolism in X‐ALD macrophages

**Figure 3:**
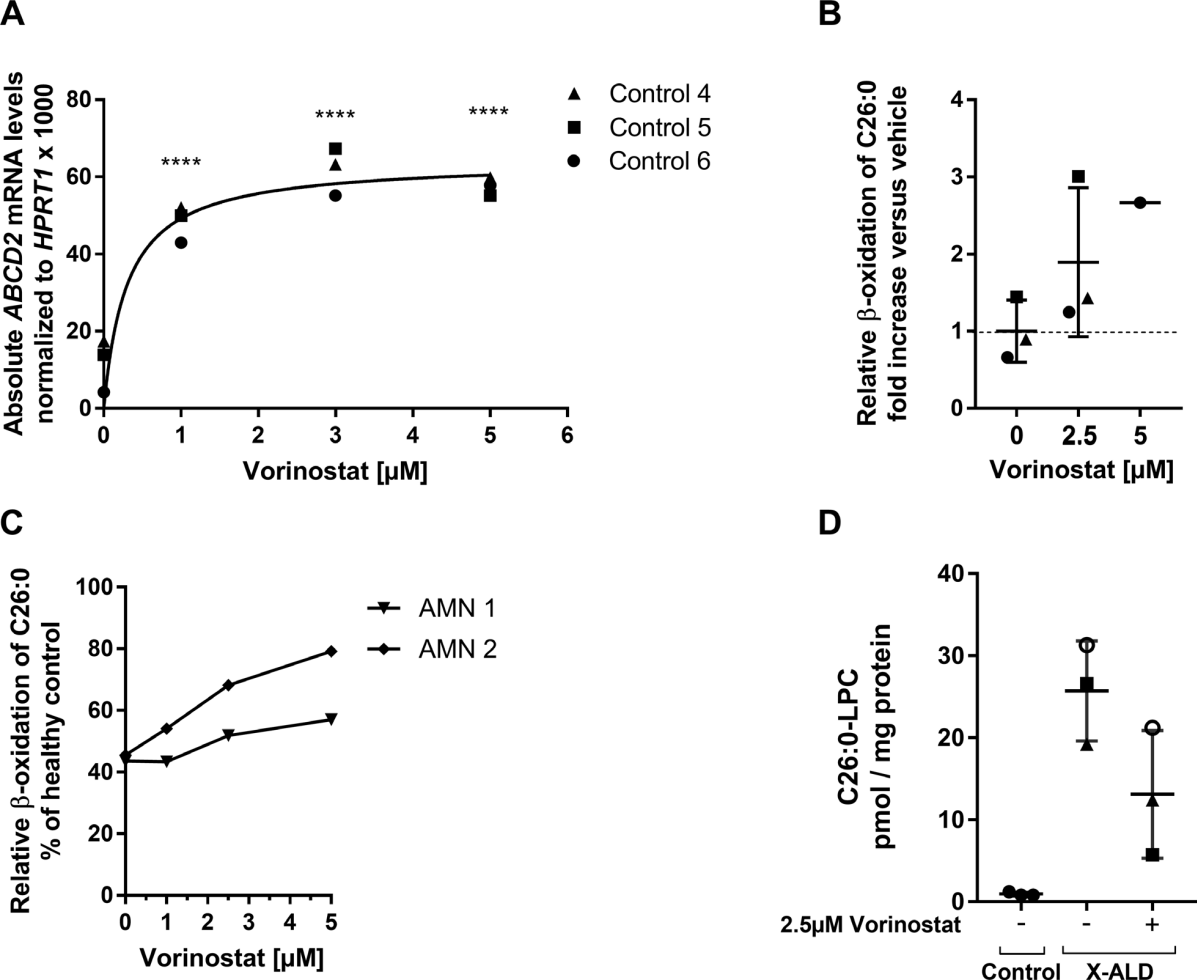
Vorinostat treatment efficiently induces *ABCD2* expression, improves *β*‐oxidation and accumulation of the VLCFA C26:0 in healthy controls and X‐ALD macrophages. (A) Macrophages (M‐CSF) from three healthy donors (Control 4, 5, and 6) were differentiated with 50 ng/mL M‐CSF for 7 days, then treated with 1, 3, and 5 *µ*mol/L Vorinostat for 24 h and absolute *ABCD2* mRNA levels were determined and normalized to *HPRT1*. (B) Macrophages from three healthy donors were differentiated as described in (A) and further activated by 100 ng/mL IL‐4 to obtain activated anti‐inflammatory macrophages. In the presence of IL‐4, macrophages were treated with 2.5 *µ*mol/L Vorinostat (*n* = 3) or 5 *µ*mol/L Vorinostat (*n* = 1) for 48 h prior to measuring C26:0 degradation levels via *β*‐oxidation. The obtained *β*‐oxidation rates for C26:0 (pmol/min/mg protein) of the added cells are depicted as fold increase versus the mean of DMSO‐treated controls (*n* = 3). (C) A dose–response curve for the *β*‐oxidation of C26:0 was generated with 1, 2.5, and 5 *µ*mol/L Vorinostat in macrophages (as described in (B)) from two X‐ALD patients (AMN 1 and AMN 2). The measured activities are shown as % of the DMSO‐treated healthy control (Control 10) value. (D) Macrophages (M‐CSF) from X‐ALD patients (*n* = 3) were stimulated with 2.5 *µ*mol/L Vorinostat for 5 days and the levels of C26:0‐LPC (pmol/ mg protein) were measured and compared to DMSO‐treated (*n* = 3) or untreated healthy control (*n* = 3) macrophages respectively. The statistical tests, one‐way ANOVA and Dunnett’s multiple comparison, were used to analyse induction of *ABCD2* in macrophages from controls (*n* = 3). ****P < 0.0001.

Our epigenetic analysis suggests that interfering with the histone modification state of the *ABCD2* gene could alter its expression in macrophages. Thus, we treated macrophages from three controls with different doses of the pan‐HDAC inhibitor Vorinostat and observed a dose‐dependent increase in *ABCD2* mRNA levels (Fig. 3A). At concentrations of >5 *µ*mol/L, we noted cytotoxic effects on differentiated macrophages in a viability assay based on Calcein AM staining (data not shown). In a dose‐dependent manner, Vorinostat treatment increased the degradation rate of C26:0 in macrophages derived from healthy controls (Fig. 3B) as well as X‐ALD patients (Fig. 3C, AMN 1 and AMN 2). Next, we asked whether the increased levels of peroxisomal *β*‐oxidation are sufficient to reduce the accumulation of VLCFA in X‐ALD macrophages. After 5 days 2.5 *µ*mol/L Vorinostat treatment, we observed a decrease in C26:0‐lysophosphatidylcholine (LPC) when compared to DMSO‐treated cells. It should be noted, however, that despite the average decrease in 50%, the C26:0‐LPC levels were still higher than those in untreated healthy control macrophages (Fig. 3D).

### Macrophage differentiation and anti‐inflammatory properties after Vorinostat treatment

**Figure 4:**
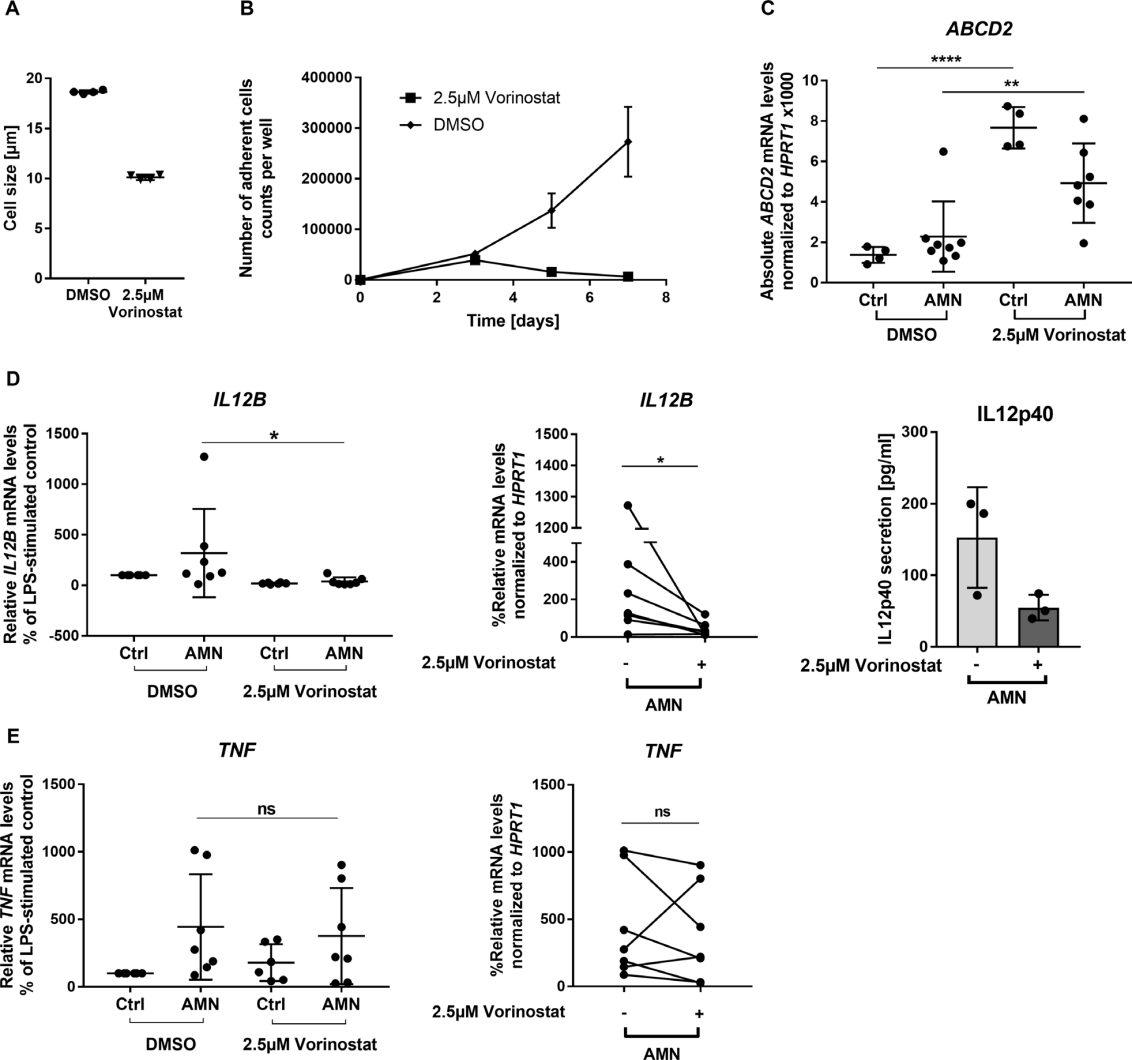
Vorinostat interferes with the differentiation of monocytes to macrophages in vitro and shows differential anti‐inflammatory effects in LPS‐activated X‐ALD macrophages. (A and B) The ability of human blood‐derived monocytes to differentiate toward macrophages when applying M‐CSF in the presence of 2.5 *µ*mol/L Vorinostat was analysed in control macrophages using two replicates from two different donors. For the differentiation analysis, we determined (A) the mean cell size after 7 days and (B) the adherence to the cell culture plate after 3, 5, and 7 days of cultivation. (C–E) To assess whether Vorinostat can efficiently reduce the elevated pro‐inflammatory gene expression of *IL12B* and *TNF* in X‐ALD, macrophages (M‐CSF) from healthy donors and X‐ALD patients were stimulated with 100 ng/mL LPS for 24 h. In the presence of LPS, macrophages (Ctrl, *n* = 4 or *n* = 6; AMN, *n* = 7) were treated with 2.5 *µ*mol/L Vorinostat or its solvent DMSO before the mRNA levels of *ABCD2* (C), and the pro‐inflammatory cytokines *IL12B* (D) and *TNF* (E) were determined by RT‐qPCR and normalized to *HPRT1*. In addition, supernatants of stimulated macrophages were harvested and IL12p40 secretion was measured by ELISA (D). The efficacy of *ABCD2* induction in control and X‐ALD macrophages was analysed by one‐way ANOVA and Sidak’s multiple comparisons test. (D and E) For comparison purposes, the levels of LPS‐treated controls were set to 100% for each experiment and the Vorinostat‐induced changes of pro‐inflammatory gene expression in X‐ALD were subjected to statistical analysis applying the nonparametric Friedman test and Dunn’s multiple comparison test. The error bars in panel (A) represent SD. *P < 0.05, **P < 0.01, ****P < 0.0001, *ns* = not significant.

Vorinostat strongly interfered with differentiation of monocytes into macrophages, as shown by a retained monocyte‐like cell size (Fig. 4A) and a reduced number of adherent cells, typical for macrophages differentiated with M‐CSF for 7 days in vitro (Fig. 4B). We previously found that X‐ALD‐derived macrophages are less able to adapt an anti‐inflammatory state after in vitro stimulation with LPS for 24 h and especially showed increased expression of *TNF* and *IL12B* in comparison to controls.^16^ Thus, we investigated how Vorinostat affects this elevated pro‐inflammatory response in X‐ALD macrophages. First, we confirmed that Vorinostat treatment induced *ABCD2* expression also in LPS‐stimulated X‐ALD and control macrophages (Fig. 4C). We did not observe significant differences in the ability of *ABCD2* induction between macrophages derived from X‐ALD patients and control cells. Vorinostat significantly reduced *IL12B* mRNA levels in macrophages derived from all seven X‐ALD patients (Fig. 4D). However, the *TNF* mRNA levels after Vorinostat treatment were not significantly reduced, although macrophages derived from five of seven X‐ALD patients showed a trend toward lower *TNF* levels (Fig. 4E). We further analysed the secretion of the cytokine IL12p40, which is encoded by *IL12B* and found lowered levels of IL12p40 in the supernatant of macrophages derived from three X‐ALD patients upon Vorinostat treatment (Fig. 4D). Together, these results show that Vorinostat abrogates repressed *ABCD2* expression in differentiated human macrophages and partially improves the impaired ability to resolve a pro‐inflammatory status of LPS‐stimulated X‐ALD macrophages.

### Vorinostat partially normalized signs of neuroinflammation in an advanced CALD patient

**Figure 5:**
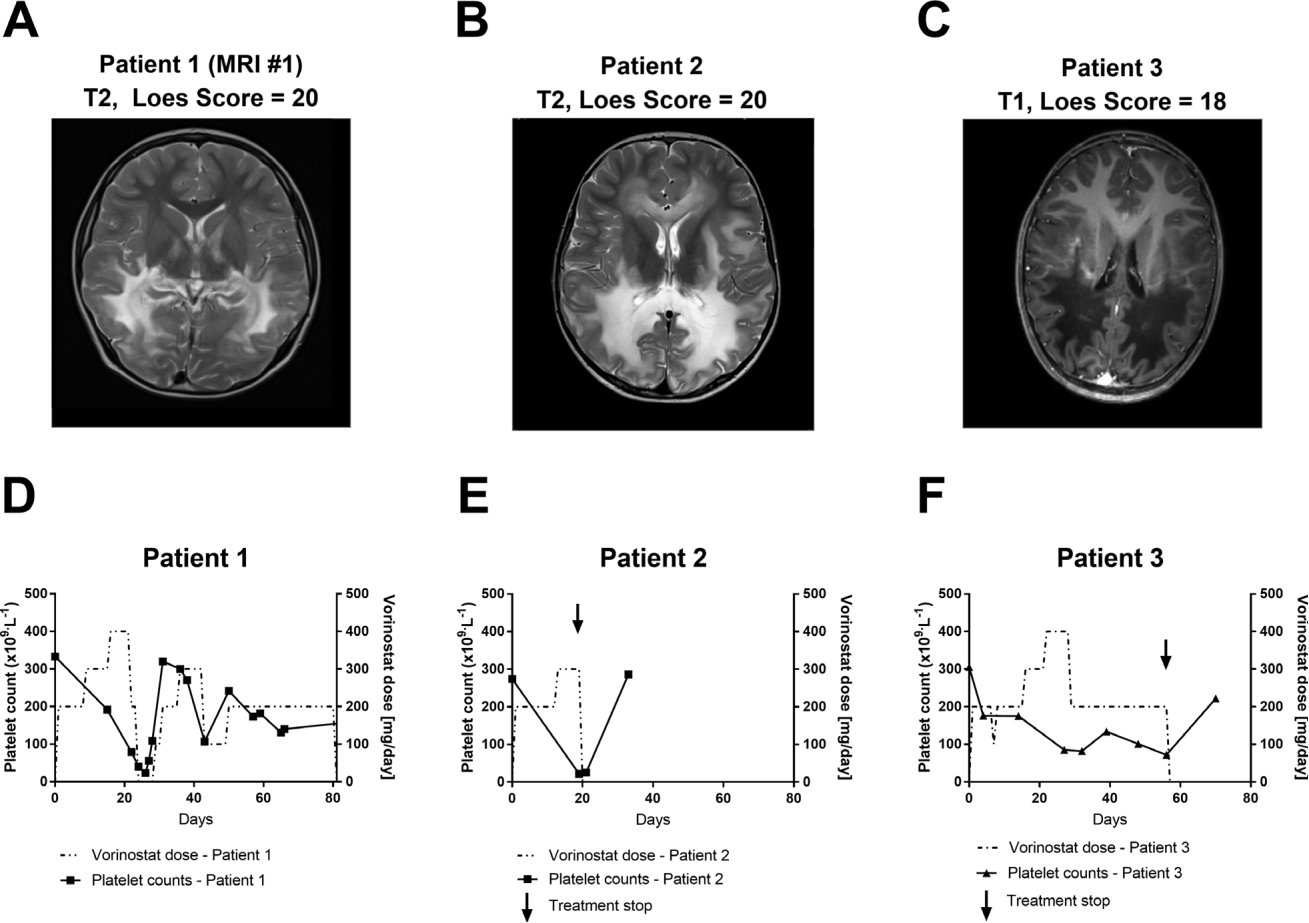
Vorinostat treatment withdrawal was required due to the severity of induced thrombocytopenia in two of three treated CALD patients who showed advanced lesions on brain MRIs. (A–C) The brain MRIs (T1‐ or T2‐weighted as indicated) obtained from three patients with advanced CALD with assigned Loes scores of 20, 20, and 18 are shown before they received 100–400 mg/day Vorinostat. (D–F) Vorinostat dose (dashed line) and platelet (thrombocyte) counts (solid line) are depicted for each patient during the treatment period. The arrow indicates the treatment stop for CALD patient 2 and 3.

Based on available knowledge together with our present findings, Vorinostat was offered to three boys diagnosed with CALD on compassionate use. In these patients, demyelination with Loes scores of 20, 20, and 18 points, respectively, (Fig. 5A–C) was too advanced for HSCT or HSCGT as worthwhile therapy (recommended at Loes scores < 9). All three received Vorinostat for up to 80 days (Fig. 5D–F). The dose‐limiting toxicity of Vorinostat was thrombocytopenia, which caused a transient reduction and pause (patient CALD1, Fig. 5D) or permanent withdrawal (patients CALD2 and CALD3, Fig. 5E and F) of Vorinostat. Patients CALD2 and CALD3 also experienced relevant gastrointestinal toxicity and patient CALD1, after >60 days of treatment, some limited hair loss.

**Figure 6:**
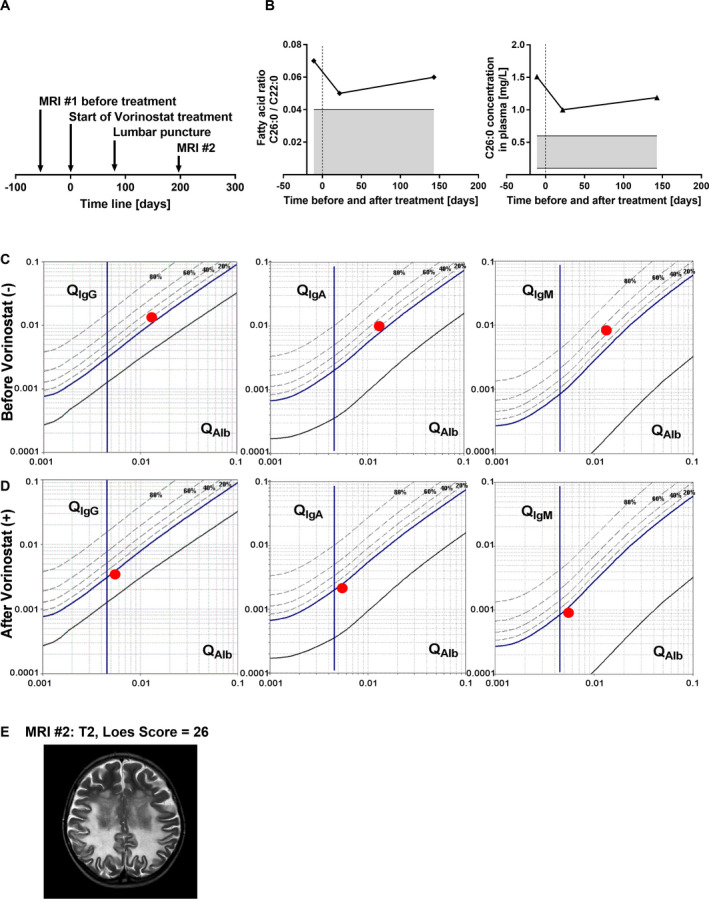
Medication with Vorinostat reversed intrathecal immunoglobulin synthesis and the disturbed BCSFB/BBB permeability but did not stop disease progression in a patient with advanced CALD. (A) As shown in the scheme, blood serum and lumbar puncture‐derived CSF were collected from patient CALD1 at the start of treatment with Vorinostat (day 0) and after 80 days of treatment and processed for analysis of albumin (Alb) and Ig levels. (B) The values of the VLCFA C26:0, measured by GC–MS, in the plasma from patient CALD1 are depicted before and during Vorinostat treatment and were normalized to the fatty acid C22:0 or are shown as mg per L plasma. The grey area indicates the normal range during physiological conditions. (C‐D) The ratios (CSF/serum level) of IgG, IgA, and IgM are plotted against the CSF/serum ratio of albumin (red dot, patient CALD1) on logarithmic scales in the Reiber Scheme, (C) before (−) and (D) after (+) Vorinostat treatment. The vertical line indicates the age‐dependent threshold for Alb permeability under physiological conditions on the x‐axis. The dashed lines indicate the percentage of intrathecal synthesis for each Ig class on the y‐axis. (E) An additional brain MRI (#2, T2‐weighted) was obtained 197 days after starting Vorinostat medication and the assigned Loes score of 26 indicated progression of the inflammatory demyelination.

Although the clinical disease status further progressed in these patients, most likely due to the advanced disease state of the patients and the short treatment period, some effects could be observed. Measurement of the VLCFA C26:0 in plasma from patient CALD1 indicated a decrease shortly after Vorinostat was started (Fig. 6A and B). Before Vorinostat treatment, the levels of total CSF protein, albumin, IgG, IgA, and IgM as well as their CSF/serum ratios were clearly increased (Table 1). This is in agreement with the observed gadolinium enhancement (not shown) along the demyelinating brain lesions (Fig. 5A, MRI#1), indicating a disruption of the BCSFB/BBB. Remarkably, after 80 days of Vorinostat treatment, the CSF levels of albumin, IgG, IgA, and IgM as well as their CSF/serum ratios were normalized (Table 1). The CSF/serum quotient of each Ig (Q_Ig_) class plotted against the CSF/serum quotient of albumin (Q_Alb_) in the Reiber scheme allows a refined interpretation of these parameters and the relative contributions of BCSFB/BBB disruption and intrathecal antibody production.^8^ The scheme for patient CALD1 before Vorinostat treatment (Fig. 6C) visualizes the BCSFB/BBB disturbance and intrathecal synthesis of all three Ig classes, while the scheme post‐treatment (Fig. 6D) shows normalization of Q_Alb_ and Q_IgG_, Q_IgA_ and Q_IgM_ implying a restored BCSFB/BBB integrity and abrogation of neuroinflammatory signs. However, a few months later an additional brain MRI revealed expansion of the demyelinating brain lesions and gadolinium enhancement (not shown) indicating ongoing BBB breakdown (Fig. 6E, MRI#2).

**Table 1:** Cerebrospinal fluid and serum levels of albumin and immunoglobulins in a patient with advanced CALD before and after Vorinostat treatment.

Vorinostat	CSF (mg/L)		Serum (g/l)			Q (CSF/Serum) ×1000		Local synthesis (%)^1^	
	−	+	−	+	Normal range	−	+	−	+
Total protein	841.1	276.4	−	−		−	−	−	−
Albumin	542.7	191.0	40.4	34.2	35–55	13.4	5.6	−	−
IgG	109.8	15.6	8.4	4.6	7–18	13.0	3.4	16.0	0.0
IgA	17.0	2.4	1.7	1.1	0.9–4.5	9.8	2.1	20.0	0.0
IgM	3.8	0.2	0.5	0.18	0.6–2.8	8.3	0.0	46.0	0.0

^1^ The local synthesis represents the intrathecal synthesis of each Ig class. The values are derived from the Reiber scheme as shown in Figure 6C and D.

**Figure 7:**
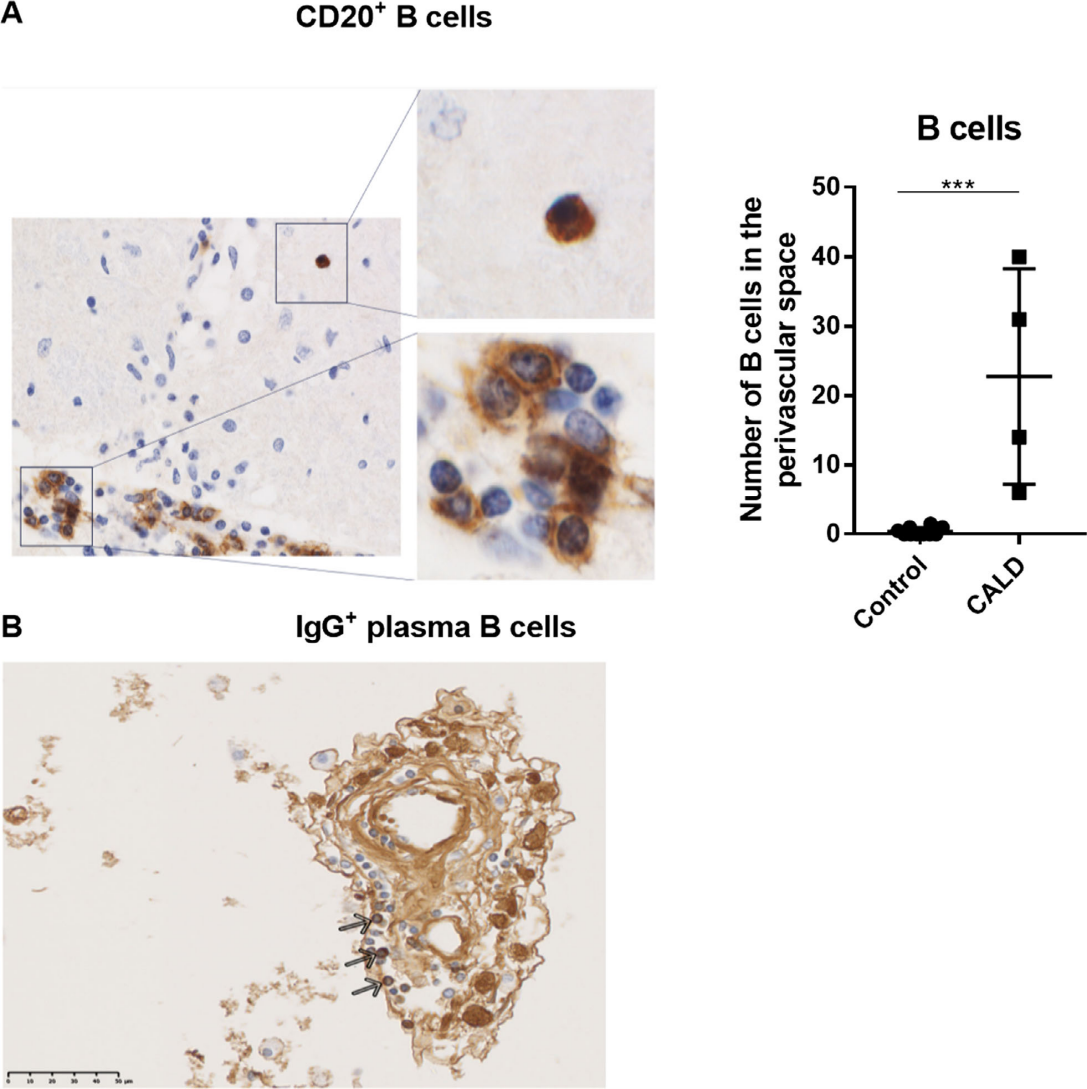
Perivascular B cell accumulation in CALD brain lesions. The light microscopy views depict immunohistochemical staining (brown) of (A) CD20+ B cells and (B) IgG+ plasma B cells (arrows) accumulating in the perivascular space in areas of active demyelinating lesions in *postmortem* brain tissue of a CALD patient. Perivascular CD20+ B cells were quantified in four untreated CALD cases (*n* = 4) and compared to controls lacking any signs of brain inflammation (*n* = 10). An occasional (very rare) parenchymal cell is shown in the magnified view (top right panel). For statistical analysis, the nonparametric Mann–Whitney test was performed. ***P < 0.001.

To further explore the origin of the observed intrathecal Ig synthesis, we analysed active demyelinating lesions in *postmortem* brain tissue derived from four CALD patients and compared those to control cases with intact brain tissue (Fig. 7). Immunohistochemistry revealed in addition to a high infiltration of monocytes/macrophages^16^ a significant recruitment and accumulation of B cells (CD20^+^) in the perivascular space (Fig. 7A), however not in the brain parenchyma. In good agreement to previous studies, staining of IgG revealed few antibody‐producing plasma cells in the perivascular cuffs in one of the four CALD cases (Fig. 7B).

## Discussion

HSCT or HSCGT can halt both inflammation and demyelination in CALD patients, but the underlying mechanism is not fully understood. However, it is believed that the predominant mechanism is mediated by donor‐derived or autologous, genetically corrected macrophages entering the inflammatory brain lesion site.^6^, ^16^ As other peripheral immune cells have no major metabolic defect in X‐ALD,^15^ a pharmacological correction of the impaired monocyte/macrophage lineage and possibly also CNS‐resident mononuclear phagocytes (including microglial cells) appears to be a promising therapeutic strategy to correct the function of mononuclear phagocytes in X‐ALD more rapidly than HSCT or HSCGT. During HSCT, it is assumed that macrophages replace nonfunctional microglial cells in the CNS. In X‐ALD, previous reports indicated a preactivated and proapoptotic phenotype of microglial cells associated with prelesional areas.^38^, ^39^, ^40^ Intriguingly, a recent study found positive effects of Vorinostat treatment on murine microglia of a stroke model.^41^ Thus, Vorinostat could be beneficial not only for monocytes/macrophages, but also for microglial cells showing metabolic and functional defects in the context of X‐ALD.

Under healthy conditions, the brain availability of Vorinostat is probably low due to clearance of the compound by BCSFB/BBB efflux transporters.^42^ In X‐ALD, however, changes have been observed in molecules associated with BBB integrity,^9^ which is further disrupted with the onset of inflammatory CALD. Therefore, in X‐ALD patients, Vorinostat may reach macrophages in the brain parenchyma at efficacious doses and the therapeutic effect may further be extended to other brain cell types.

Based on our epigenetic analysis of the *ABCD2* promotor, we focused on HDAC inhibitors to functionally rescue macrophages in X‐ALD. We specifically selected Vorinostat because of its property to metabolically and immunologically improve X‐ALD macrophages, which is essential for efficient clearance of myelin debris enriched in VLCFAs and, thus, contributing to an anti‐inflammatory milieu in the CALD lesions.^43^ In vitro, Vorinostat lowered the secretion of the pro‐inflammatory cytokine IL12p40 in LPS‐activated X‐ALD macrophages, which is a chemoattractant mainly produced by monocytes/macrophages and regulates T cell responses.^44^ In contrast, Vorinostat showed dose‐dependent effects on *TNF* expression, indicating that Vorinostat is not solely an anti‐inflammatory compound but depending on the dosage, has differential properties in human X‐ALD derived macrophages. In addition, Vorinostat interferes with the differentiation of monocyte‐derived cells^24^ like macrophages (cf. Fig. 4), thus possibly directly lowering the number of activated monocyte‐derived macrophages entering the CALD brain parenchyma and thereby additionally reducing local proinflammatory cytokine secretion.

Based on the available data, three advanced CALD patients, not eligible for HSCT/HSCGT received compassionate Vorinostat treatment. Due to the progressed disease state of these patients, the primary goal of this off‐label application was to assess the potential of Vorinostat to interfere with disease progression, using the correction of the BCSFB/BBB integrity as a positive readout. The assessment of the CSF/serum Ig and albumin quotients demonstrated a normalization of the BCSFB/BBB integrity in patient CALD1 comparable to neurological asymptomatic X‐ALD or arrested atypical CALD patients.^8^ This observation fits a previous report showing that Vorinostat positively affects the integrity of the blood‐retinal barrier in experimental autoimmune uveitis mice.^45^ Three months after performing the CSF diagnostics and 3 weeks after Vorinostat discontinuation, brain MRI of patient CALD1 showed expansion of the demyelinating lesions and gadolinium enhancement. Several reasons could account for these two observations. The tightening of the BCSFB/BBB permeability after Vorinostat application might have been timely connected to Vorinostat intake and thus, might have been lost after discontinuation of the treatment. The improvement of the BCSFB/BBB permeability could also represent a temporary tightening, similar to that recently observed, although to a much lower extent, after HSCGT intervention.^12^ Alternatively, the correction might have been partial, thus preventing the infiltration of larger molecules such as albumin and Igs but not of low‐MW molecules like gadolinium. In addition, the properties of the BBB as a true barrier and the BCSFB as an “educational gate” might be related to the observed disparate effects by Vorinostat.^46^ Accordingly, despite the partial/temporary improvement of the BCSFB/BBB permeability, Vorinostat treatment did not halt the far advanced white matter destruction in this CALD patient. Thus, although Vorinostat seems to promptly improve the tightness of the BCSFB/BBB, beneficial effects on myelin loss may require treatment commencement at earlier disease stages.

We conclude that Vorinostat treatment may have beneficial effects on BCSFB/BBB integrity in CALD patients. However, like for HSCT/HSCGT, also a pharmacological treatment with Vorinostat seems to require a disease state that is not too far advanced. Based on these results, it is not possible to predict a positive outcome even when treatment is started at an earlier disease stage. All three CALD patients developed severe thrombocytopenia and, thus, a reduced drug intake or even an early treatment withdrawal was required. Currently, more selective HDAC inhibitors, specifically targeting class I HDACs are investigated in preclinical and clinical trials for cancer, inflammatory, and neurodegenerative diseases. Due to lower cytotoxicity with maintained anti‐inflammatory and neuroprotective effects, some of these compounds might be more suitable for CALD treatment than Vorinostat.^47^ In particular, monocyte/macrophage‐selective HDAC inhibitors sparing major side effects like thrombocytopenia are highly attractive candidates for further investigations.^48^, ^49^ In summary, a pharmacological treatment without severe side effects, that positively affects the BCSFB/BBB and possibly ameliorates the inflammatory demyelination in CALD patients, would not necessarily be restricted to patients uneligible for HSCT or HSCGT but could represent a sequential treatment option to bridge the time gap between diagnosis and other interventions.

## Conflict of Interest

We declare that we have no competing interests.

## Author Contributions

Conceptualization: J Berger, I Weinhofer, and B Zierfuss; Methodology: I Weinhofer, B Zierfuss, JS Kühl, W Köhler, A Bley, K Zauner, J Binder, K Martinović, C Seiser, C Hertzberg, S Kemp, G Egger, G Leitner, J Bauer, C Wiesinger and M Kunze; Investigations: I Weinhofer, B Zierfuss, JS Kühl, W Köhler, A Bley, K Zauner, J Binder, K Martinović, C Seiser, C Hertzberg, S Kemp, G Egger, G Leitner, J Bauer, C Wiesinger, and M Kunze; Writing: I Weinhofer, B Zierfuss, J Berger, and S Forss‐Petter; Funding acquisition: J Berger, C Wiesinger.

## Supporting information


**Table S1.** Description of controls and X‐ALD patients.
