# Supplementary material for: Vorinostat in the acute neuroinflammatory form of X‐linked adrenoleukodystrophy
Source: Ann Clin Transl Neurol. 2020 May 2;7(5):639–52. doi: 10.1002/acn3.51015 (PMC7261758; doi:10.1002/acn3.51015)
Supplement: Supplementary file 1 — Table S1. Description of controls and X‐ALD patients. [file ACN3-7-639-s001.docx]

Supplementary Table 1: Description of controls and X-ALD patients

| **Proband ID** | **Age** | **Sex** | **Ethnicity** | **Steroid hormone replacement therapy** ^§§^ |
| --- | --- | --- | --- | --- |
| Control 1 | 30 | m | n.a. | - |
| Control 2 | <35 | m | n.a. | - |
| Control 3 | <35 | m | n.a. | - |
| Control 4 | n.a. | m | n.a. | - |
| Control 5 | 53 | m | n.a. | - |
| Control 6 | n.a. | m | n.a. | - |
| Control 7 | 29 | m | n.a. | - |
| Control 8 | 54 | m | n.a. | - |
| Control 9 | 57 | m | n.a. | - |
| Control 10 | 26 | m | Caucasian | - |
| Control 11 | 49 | f | n.a. | - |
| Control 12 | 42 | f | n.a. | - |
| Control 13 | 44 | m | Caucasian | - |
| Control 14 | 26 | m | Caucasian | - |
| Control 15 | 24 | m | Caucasian | - |
| Control 16 | 54 | m | Caucasian | - |
| Control 17 | 26 | m | Caucasian | - |
| Control 18 | 29 | m | Caucasian | - |
| AMN 1 | 44 | m | Caucasian | + |
| AMN 2a, 2b^§^ | 26 | m | Caucasian | + |
| AMN 3 | 38 | m | Caucasian | - |
| AMN 4 | 31 | m | Caucasian | + |
| AMN 5 | 41 | m | Caucasian | - |
| AMN 6 | 36 | m | Caucasian | + |
| AMN 7 | 44 | m | Caucasian | - |
| CALD 1 | 8 | m | Caucasian | + |
| CALD 2 | 7 | m | Caucasian | + |
| CALD 3 | 10 | m | Caucasian | + |

| ^§^ Patient AMN 2 donated blood twice within 1 year, indicated as AMN 2a and AMN 2b | |
| --- | --- |
| ^§§^ Four AMN and three CALD patients received steroid hormone replacement (+) to treat adrenal insufficiency | |
| Abbreviations: m, male; f, female; n.a., data not available |  |
